# Knife-assisted full-thickness resection guided by pocket detection method for detection and complete excision of deeply invasive rectal cancer

**DOI:** 10.1016/j.vgie.2024.11.004

**Published:** 2024-11-23

**Authors:** Maria Eva Argenziano, Andrea Sorge, Pieter Jan Poortmans, Michele Montori, Daniele Balducci, Anne Hoorens, Luca Maroni, David James Tate

**Affiliations:** 1 Clinic of Gastroenterology, Hepatology and Emergency Digestive Endoscopy, Università Politecnica delle Marche, Ancona, Italy; 2 Department of Gastroenterology & Hepatology, University Hospital Ghent (UZ Gent), Gent, Belgium; 3 Faculty of Medicine and Health Sciences, University of Gent, Gent, Belgium; 4 Department of Pathophysiology and Transplantation, University of Milan, Milan, Italy; 5 Department of Gastroenterology & Hepatology, University Hospital Brussels (UZ Brussels), Brussels, Belgium; 6 Department of Anatomopathology, University Hospital Ghent (UZ Gent), Gent, Belgium

A 61-year-old patient with concomitant esophageal (cT2N0M0) and pharyngeal (cT3N2M0) squamous cell cancers under systemic treatment (Charlson Comorbidity Index 4) was referred for a rectal lesion detected on a positron emission tomography scan. Endoscopy showed a sessile 25-mm proximal rectal lesion with a demarcated and slightly depressed central area (Paris 0-Is+IIc, Japan Narrow-Band Imaging Expert Team-3) on the posterior wall (Fig. 1A). Magnetic resonance imaging ruled out extramural vascular and nodal involvement and confirmed the extraperitoneal location of the lesion. EUS raised suspicion of muscularis propria involvement. Considering the patient’s active oncologic comorbidities, an endoscopic resection was suggested after a multidisciplinary team (MDT) discussion. Device-assisted endoscopic full-thickness resection was not feasible due to lesion size.^1^

**Figure 1:**
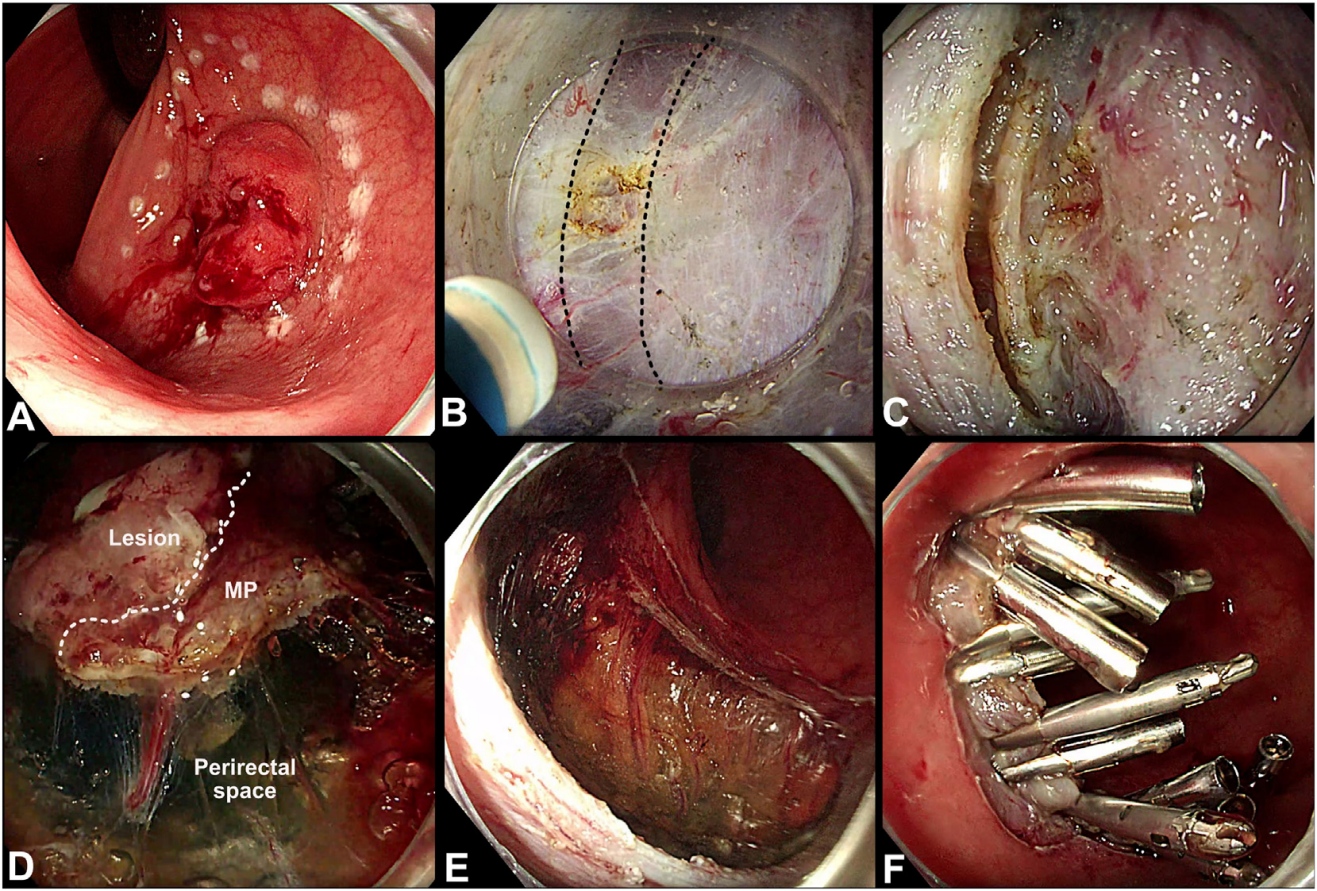
**A,** The large nonpedunculated colorectal polyp from the study located in the proximal rectum with a depressed central area (Paris 0-Is+c, JNET-3). **B,** Endoscopic image of the suspected muscle-retracting sign (MRS) within the submucosal tunnel. **C,** First muscularis propria (MP) incision 3 mm caudal to the MRS. **D,** Completed circumferential muscular incision. **E,** Defect after removal of the lesion. **F,** Defect closure with through-the-scope clips.

A submucosal pocket was created 15 mm away from the lesion in the direction of the suspected deeply invasive component (SIC), namely the area of the maximally disrupted vascular pattern (Video 1, available online at www.videogie.org). A muscle-retracting sign, namely the tethering of the muscularis propria to the overlying mucosa suggestive of deep submucosal invasion, was revealed using the saline immersion technique^2^ (Figs. 1B and 2). Thus, a circular mucosal incision was made, followed by a submucosal dissection to isolate the SIC. Traction using a multiband device and a surgical wire was applied to enhance access to the resection plane. A knife-assisted incision of the muscularis propria was performed circumferentially around the SIC at a distance of 3 mm (Fig. 1C). The incision was performed using Dry Cut (Effect 3) (VIO 3; ERBE Elektromedizin, Tübingen, Germany). The knife-assisted full-thickness resection (kFTR) was completed by dissecting the muscularis propria from the perirectal fat (Fig. 1D). Careful inspection of the postresection defect was performed, and bleeding vessels were treated (Fig. 1E). The muscular defect was completely closed with through-the-scope clips (Fig. 1F). A 5-day course of empiric antibiotics was administered.

**Figure 2:**
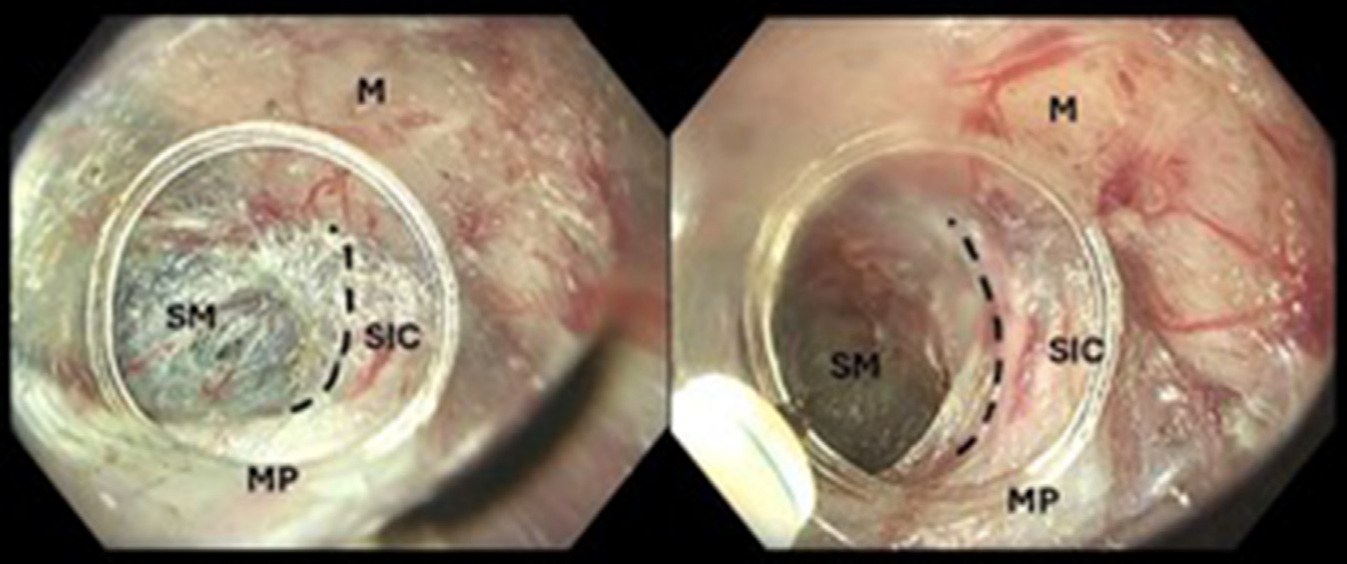
The appearance of the suspected deeply invasive component (SIC) within the tunnel. Tethering of the mucosal layer (specimen) (M) to the muscularis propria (MP) on the right side of the images is demonstrated; a *dotted black line* indicates demarcation of the SIC from the standard submucosal appearance. *M*, Mucosa; *SM*, submucosa.

The postprocedural course was uneventful, and the patient was discharged home 24 hours after the resection. Histopathology revealed an R0 resection of a low-grade intestinal-type adenocarcinoma with a deeply submucosally invasive component in contact with the muscularis propria, without tumor budding or perineural invasion (pT1b, Kikuchi level sm3). Focal lymph vascular invasion was observed (Fig. 3). Given the radical resection and the patient’s clinical status, the MDT and the patient agreed on endoscopic and radiologic follow-up without further treatment. A 3-month follow-up CT scan revealed neither nodal nor distal metastases. A sigmoidoscopy performed 6 months after the resection showed no local recurrence or long-term adverse events. The patient remained asymptomatic, with no functional impairment related to the resection.

**Figure 3:**
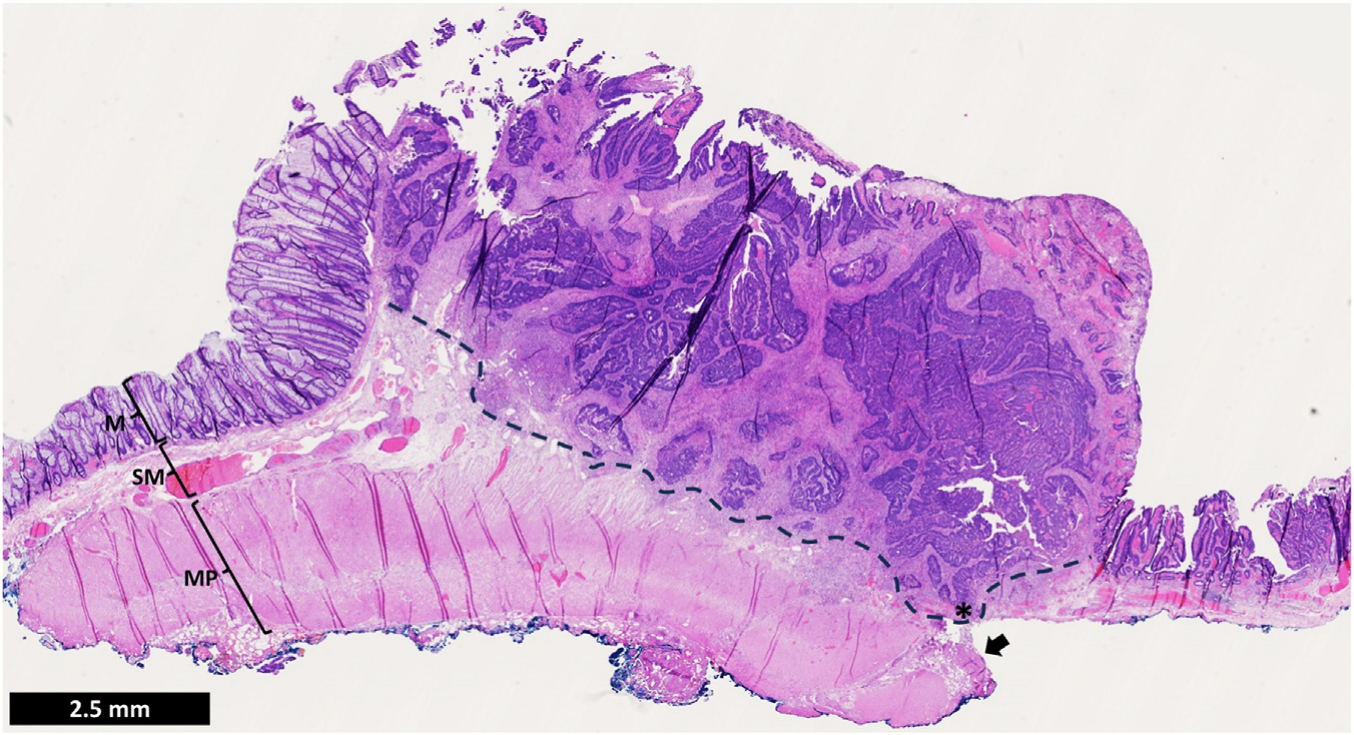
Histopathology H&E overview (orig. mag. ×8). The *dashed line* marks the invasive front of the tumor, and the *asterisk* marks the deepest point of invasion, which is deep in the submucosa (sm3), just above the muscularis propria (MP). The *arrow* marks an area where the MP is slightly detached from the submucosa, and when the muscle fibers contract after excision, the muscle layer is pulled down slightly. *M*, Mucosa; *SM*, submucosa.

Initial reports of exposed kFTR have been described for the excision of severely fibrotic or deeply invasive lesions.^3^, ^4^, ^5^ In this case, kFTR guided by the pocket detection method provided a feasible and safe approach for precise detection and radical resection of a deeply invasive cancer in the posterior rectal wall in a patient unfit for surgery. This novel endoscopic technique may represent an alternative to surgical local excision strategies (eg, transanal minimally invasive surgery and transanal endoscopic microsurgery) in the posterior rectum in carefully selected cases managed at expert centers.

## Disclosure

Dr Tate is a consultant for and has received research funding from Olympus, Fujifilm, and Pentax. The other authors disclosed no financial relationships.

## Supplementary data

Video 1 Tunneling knife-assisted full-thickness resection technique.
